# Settling on leaves or flowers: herbivore feeding site determines the outcome of indirect interactions between herbivores and pollinators

**DOI:** 10.1007/s00442-019-04539-1

**Published:** 2019-11-04

**Authors:** Quint Rusman, Peter N. Karssemeijer, Dani Lucas-Barbosa, Erik H. Poelman

**Affiliations:** 1 Laboratory of Entomology, Wageningen University, P.O. Box 16, 6700 AA Wageningen, The Netherlands; 2 Present Address: Bio-communication and Ecology, ETH Zürich, Schmelzbergstrasse 9, 8092 Zurich, Switzerland

**Keywords:** Antagonist-mutualist interactions, Florivory, Folivory, Plant defense, Plant-mediated interactions, Preference–performance

## Abstract

Herbivore attack can alter plant interactions with pollinators, ranging from reduced to enhanced pollinator visitation. The direction and strength of effects of herbivory on pollinator visitation could be contingent on the type of plant tissue or organ attacked by herbivores, but this has seldom been tested experimentally. We investigated the effect of variation in feeding site of herbivorous insects on the visitation by insect pollinators on flowering *Brassica nigra* plants. We placed herbivores on either leaves or flowers, and recorded the responses of two pollinator species when visiting flowers. Our results show that variation in herbivore feeding site has profound impact on the outcome of herbivore–pollinator interactions. Herbivores feeding on flowers had consistent positive effects on pollinator visitation, whereas herbivores feeding on leaves did not. Herbivores themselves preferred to feed on flowers, and mostly performed best on flowers. We conclude that herbivore feeding site choice can profoundly affect herbivore–pollinator interactions and feeding site thereby makes for an important herbivore trait that can determine the linkage between antagonistic and mutualistic networks.

**Electronic supplementary material:**

The online version of this article (10.1007/s00442-019-04539-1) contains supplementary material, which is available to authorized users.

## Introduction

Flowering plants are attacked by herbivores and at the same time, interact with pollinators for reproduction. When herbivores attack a plant, this not only affects plant growth and survival (Züst and Agrawal 2017), but can also affect plant interactions with pollinators and consequently seed production (Rusman et al. 2019a). The outcome of herbivore–pollinator interactions can vary tremendously: herbivory can have negative, positive, or no effects on pollinator visitation (Kessler and Halitschke 2009; Moreira et al. 2019; Rusman et al. 2019b). In extreme cases, herbivory may lead to a shift in the principal pollinator of plants under attack (Kessler et al. 2010). Moreover, herbivore-induced changes in pollinator behavior can lead to changes in pollinator community composition with consequences for plant reproductive success (Chautá et al. 2017; Hoffmeister et al. 2016; Rusman et al. 2018). Despite the apparent commonness of herbivore–pollinator interactions (Moreira et al. 2019), and their potential impact on plant ecology and evolution (Rusman et al. 2019a), we known surprisingly little about the causes of variability observed for these interactions.

Herbivores can directly or indirectly influence plant attractiveness to pollinators. Direct herbivore–pollinator interactions occur when the presence of the herbivore itself alters pollinator visitation. For example, pollinators may be repelled by herbivores on flowers, because they can hinder access to the flower (Lohman et al. 1996), signal an increased predation pressure (Moreira et al. 2019), or interrupt pollinators during feeding (Canela and Sazima 2003). In contrast, inflorescences with herbivores like aphids may be attractive due to the secretion of honeydew (Almohamad et al. 2009; Meiners et al. 2017). Indirect herbivore–pollinator interactions occur through an intermediary species (Wootton 1994), often the plant. Plants use a variety of flower traits to attract pollinators such as bright colors, complex scent mixtures, and rewards in the form of nectar and pollen (Junker and Parachnowitsch 2015). These traits can readily change upon herbivore attack (Chautá et al. 2017; Rusman et al. 2019b; Schiestl et al. 2014). Such herbivore-induced changes in flower traits can negatively or positively alter plant interactions with pollinators through trait-mediated indirect interactions (Kessler et al. 2011; Ohgushi 2005; Rusman et al. 2019a). Alternatively, herbivores can alter the perception of flower traits by pollinators, without changing the trait itself, through interaction modification (Wootton 1994). For example, large colonies of black, white, or green aphids in flower heads might enhance its visual appearance by increasing the contrast of the flowers with the background. The importance of direct and indirect effects in herbivore–pollinator interactions may depend primarily on the feeding behavior of the herbivore.

Effects of herbivores on pollinator visitation differ depending on the type of plant tissue or organ attacked by the herbivores. Herbivores span a range of feeding behaviors and may preferably or exclusively attack roots, leaves, or flowers of flowering plants. Indeed, while some herbivores are obligate folivores or florivores, others move from leaves to flowers at some point in time (Agerbirk et al. 2010; Bandeili and Müller 2010; Lucas-Barbosa et al. 2013). About a decade ago, it was hypothesized that variation in herbivore feeding behavior, and especially the feeding site of the herbivore, determines the outcome of herbivore–pollinator interactions (Kessler and Halitschke 2009). Herbivores feeding on different plant parts differ in the way they affect interactions with pollinators, and induce different plant responses which extent to flower trait expression (Farré-Armengol et al. 2015; Rusman et al. 2019a, b). The feeding site of herbivores seems indeed important for the outcome of herbivore–pollinator interactions. By comparing a large number of studies, a recent meta-analysis showed overall negative effects of florivores on pollinator visitation, while folivores had only marginal negative effects, and root herbivores had no effects on pollinator visitation (Moreira et al. (2019). However, most of these previous studies investigated single herbivore species that feed on a single tissue type, and addressed especially chewing herbivores and their effects on bee pollinators. Herbivores that feed on different plant tissues offer an opportunity to experimentally manipulate herbivore feeding site and investigate its effects on different pollinators.

In this study, we tested whether herbivores feeding on leaves or flowers of black mustard affect interactions with pollinators. We specifically studied (1) how the behavior of two pollinator species, the butterfly *Pieris brassicae* and the syrphid fly *Episyrphus balteatus,* were affected by plant exposure to different herbivores, (2) if herbivore-induced changes in pollinator behavior were determined by feeding site of the herbivores on leaves or flowers, (3) if the three herbivore species—the aphids *Brevicoryne brassicae, Lipaphis erysimi*, and *Myzus persicae*—had a preference for leaves or flowers, and (4) how feeding site affected the performance of the herbivores themselves. We show that the outcome of indirect plant-mediated interactions between herbivores and pollinators is largely determined by the feeding site of the herbivore: herbivores feeding on flowers had a consistent positive effect on the attraction of different pollinators, whereas herbivores feeding on leaves did not. The evolution of herbivore feeding behavior may thus be important for pollinator network assembly via trait-mediated interactions.

## Materials and methods

### Plant and insects

Black mustard (*Brassica nigra*) seeds (accession CGN06619) were obtained from the Centre for Genetic Resources (CGN, Wageningen, The Netherlands) and propagated by open pollination in the field. Seeds were germinated in trays and 1-week-old plants were transplanted and cultivated in pots (∅ 17 cm – 2 L) filled with potting soil (Lentse potgrond) and sand in a 1:1 volume ratio under greenhouse conditions (23 ± 2 °C, 50–70% r.h., L16:D8). Plants were used in the experiments once they started flowering (5/6 weeks old).

We used three aphid species for the experiments: *B. brassicae*, *L. erysimi*, and *M. persicae*. In nature, the three aphid species are found on leaves and flowers of *B. nigra*, and based on field observations, we expected different species to prefer to build up colonies on different parts of the plant (pers. obs. Quint Rusman, Lucille Chrétien, Daan Mertens). This allowed us to compare how variation in feeding preference of species with similar feeding mode affects plant interactions with pollinators. Aphids were originally collected in the surroundings of Wageningen (The Netherlands), and are routinely reared in the Laboratory of Entomology (Wageningen University) under greenhouse conditions (22 ± 1 °C, 50–70% r.h., L16:D8). *Brevicoryne brassicae* was reared on Brussels Sprouts plants (*Brassica oleracea* variety *gemmifera* cultivar Cyrus); *L. erysimi* and *M. persicae* were reared on *Raphanus sativus*. We used two pollinator species for our behavioral experiments in the greenhouse: The butterfly *P. brassicae* and the syrphid fly *E. balteatus*. Although honeybees are the most abundant pollinators in this study system, we have shown that honeybees generally do not respond to herbivore-induced plant responses (Lucas-Barbosa et al. 2013; Rusman et al. 2018). Furthermore, behavioral responses of social pollinators such as honeybees and bumblebees should ideally not be tested in small enclosed spaces such as greenhouse compartments, because such conditions can change the behavior of these social pollinators, and would, therefore, make whatever response to choices offered unreliable (pers. obs. Quint Rusman and Dani Lucas-Barbosa). We choose to conduct our experiments in the greenhouse, because our questions are not easily addressed in the field. In a field experiment, we would need to prevent natural colonization of about 30 species of herbivorous insects that colonize *B. nigra* on leaves, flowers, or both, while still allowing pollinators to access the flowers. This is extremely difficult, with a high risk of contamination of flower-feeding herbivores in our leaf-feeding-only treatment, and leaf-feeding herbivores in our flower-feeding-only treatment (see below). *Episyrphus balteatus* is a common flower visitor and efficient pollinator of Brassicaceae (Jauker and Wolters 2008), while *P. brassicae* has a low visitation frequency on *B. nigra* compared to other pollinators in the field (Lucas-Barbosa et al. 2013; Rusman et al. 2018), but might nonetheless be important for long-distance pollen dispersal (Courtney et al. 1982). *Pieris brassicae* are routinely reared at the Laboratory of Entomology (Wageningen University) under greenhouse conditions (22 ± 1 °C, 50–70% r.h., L16:D8). Larvae were reared on Brussels Sprouts plants (*B. oleracea* variety *gemmifera* cultivar Cyrus) and adult butterflies were given a honey solution (10%). *Episyrphus balteatus* were obtained as pupae from Katz Biotech AG, Barutch, Germany. Once adult syrphid flies eclosed, they were provided with sugar, pollen, water and a Brussels Sprouts plant infested with *B. brassicae* aphids until the experiment.

### Effect of herbivore feeding site on pollinator behavior

To investigate if pollinator behavior was influenced by the feeding site of the herbivore, we recorded the behavior of two pollinator species, the butterfly *P. brassicae*, and the syrphid fly *E. balteatus*, in two-choice situations (Rusman et al. 2019b). Individual pollinators were offered a choice between an uninfested plant and a plant infested with one of the herbivores, on either leaves or flowers (see *Herbivore performance on leaves or flowers*). A single butterfly or syrphid fly was released at a time, and at 100 cm from the plants. Each individual insect was observed for 12 min. We recorded first choice for one of the two plants, the duration of the visitation, and number of flowers visited for each of the two plants. First choice was defined as the plant the insect had first contact with, either with a leaf or flower. First contact with a leaf was included, because these only comprised a small number of cases, and were always followed by movement of the pollinator to the flowers of that same plant. If the pollinator did not make a choice within 5 min, it was recorded as ‘no response’, and the observation ended. Observations were performed using a handheld computer (Psion Workabout Pro^tm^ 3, London, UK) programmed with The Observer XT software (version 10, Noldus Information Technology, Wageningen, The Netherlands). Each insect was used only once. Butterflies were used for the experiments at 2–3 days after mating and 3–10 days since eclosion from pupae. They were starved for about 20 h prior to the bioassay and provided with a Brussels Sprouts plant to lay eggs so that the observed behavior was driven by searching for food, and not for oviposition sites. Syrphid flies were 5–15 days old since eclosion, starved for 4–8 h before the experiment, and provided with a Brussels Sprouts plant infested with *B. brassicae* to lay eggs and some water to prevent dehydration. For each plant pair, 10–20 insects were tested. If more than ten insects were non-responsive, observations were terminated that day. For each plant treatment, 10–11 plant pairs were tested. Experiments were carried out in a flight chamber set-up (gauze tent of 293 cm × 200 cm × 230 cm), in a greenhouse compartment (25 ± 1 °C, 50–70% r.h., L16:D8), from the end of September (2016) till the beginning of March (2017).

### Herbivore preference for leaves or flowers

To investigate if aphids have a preference for vegetative or flowering plant tissues, we recorded feeding site chosen by winged aphids on *B. nigra* plants. A flowering plant was placed in a mesh tent (95 × 95 × 190 cm) where 20 winged aphids of one of the three species were released—*B. brassicae*, *L. erysimi*, or *M. persicae.* The winged aphids were placed in a Petri dish (diameter 9 cm) on top of a wooden pedestal (height 38 cm); this pedestal stood at approximately 50 cm from the flowering plant. Aphids had 24 h to make a choice between vegetative (young leaves, old leaves and stems) and inflorescence tissues (buds, flowers, bracts and floral stems). Aphids recorded elsewhere in the tent than on the plant were considered unresponsive. Experiments were carried out in a greenhouse compartment (23 ± 1 °C, 50–70% r.h., L16:D8) from the beginning of October (2016) till the beginning of November (2016), and for each aphid species, feeding site preference was tested for 15 plants.

### Herbivore performance on leaves or flowers

To investigate on which tissues aphids perform best, flowering *B. nigra* plants were infested with *B. brassicae*, *L. erysimi*, or *M. persicae*, on either leaves or flowers. We placed 20 adult female aphids on either 2 true leaves, 10 per leaf, or on 4 inflorescences, 5 per inflorescence, on the final inflorescences of the 4 top flowering branches. To prevent aphids from moving between vegetative and flowering parts of the plant, we attached cotton wool with a small piece of a wire around the main stem between the vegetative and flowering part of the plant. The number of aphids was recorded 7 days after infestation as a proxy of performance. For the first six plants used in the experiment, the number of aphids was both counted and estimated. The number of aphids was estimated by counting groups of 10–20 aphids rather than each individual aphid. Since estimations closely matched counting while significantly reducing counting time, only estimations were used to assess the total number of aphids per plant for the remainder of the experiment. Experiments were carried out in a greenhouse compartment (23 ± 1 °C, 50–70% r.h., L16:D8) from the end of September (2016) till the end of February (2017), and we had 25–28 plants per treatment. After assessing aphid performance, plants were used in the pollinator behavior experiment.

### Statistical analysis

For pollinator behavior data (number of insects, flowers visited, time spent per plant, and flower), we used the proportion of the response variable between infested and uninfested plants (Rusman et al. 2019b). We used generalized linear mixed models with a Poisson distribution and a log link function. The response variable was fitted to the intercept, and *plant pair* was used as a random factor. We did not correct for flower abundance in these analyses, because herbivory by two out of the three species tested in this study did not affect flower abundance in a previous study (Rusman et al. 2019b). For aphid preference and performance, we used generalized linear models with a Poisson distribution and a log link function or negative binomial distribution with a log link function to correct for overdispersion. Herbivore species, feeding site, and the interaction between herbivore species and feeding site were included in the model as fixed factors. Interactions were removed from the model if they were statistically non-significant (*P* > 0.05). For post hoc analysis, we used Tukey’s post hoc tests. For aphid preference, we corrected for the number of unresponsive aphids by including total number of aphids (responsive + unresponsive) as an offset. We used the lme4 (Bates et al. 2015), multcomp (Hothorn et al. 2008), and lmtest (Zeileis and Hothorn 2002) packages for these analyses. For correlations between numbers of aphids and visitation parameters of pollinators, we computed the correlation coefficient using the *Pearson* or *Kendall* method, depending on the distribution of the data. All analyses were carried out in R (version 3.4.3 × 64, 2017, The R Foundation for Statistical Computing Platform).

## Results

### Effect of herbivore feeding site on pollinator behavior

**Fig. 1:**
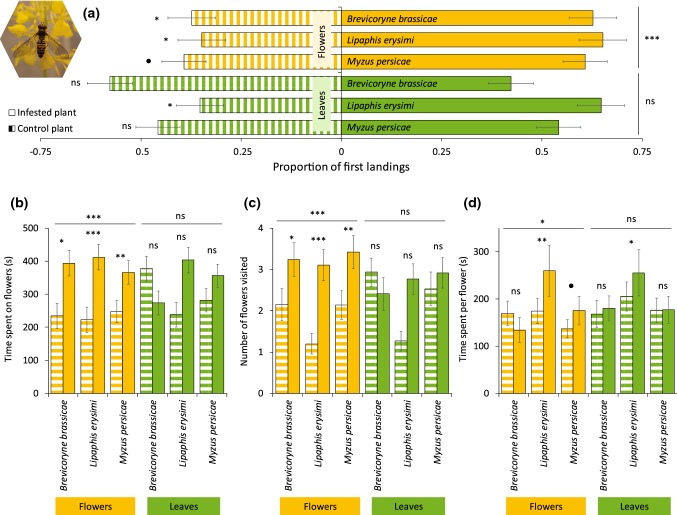
Preference of the syrphid fly *Episyrphus balteatus* for uninfested *Brassica nigra* plants or plants infested with herbivores on leaves or flowers. **a** Proportion of syrphid flies (Mean ± SE) that first landed on flowers or leaves of *B. nigra* plants infested with herbivores on leaves or flowers, or uninfested plants. **b** Visitation time (Mean ± SE), **c** number of flowers visited (Mean ± SE), (**d**) and time spent per flower (Mean ± SE) by individual pollinators on infested or uninfested *B. nigra* plants. Number of replicates per herbivore treatment varied between 66 and 83 syrphid flies, and 7 and 9 plant pairs. Asterisks above bars indicate significant differences with *** = *P* < 0.001, ** = 0.001 ≥ *P* < 0.01, * = 0.01 ≥ *P *≤ 0.05, and ● = 0.05 > *P* < 0.1, based on Tukey’s post hoc tests. Picture shows an *E. balteatus* syrphid fly visiting flowers of *B. nigra*. Photograph credits: Quint Rusman

We observed the behavior of 908 responsive pollinators, with 182 h of observation time, and about 9000 flower visits. The behavior of syrphid flies and butterflies was influenced by herbivore infestation, and the effects depended on herbivore and pollinator species, as well as the feeding site of the herbivore. Overall, syrphid flies landed more frequently on flower-infested plants when compared with uninfested plants (Fig. 1, GLMM: *z* = 3.67, *P *< 0.001). This was true for plants infested with *L. erysimi* (GLMM: *z* = 2.42, *P *= 0.015), *B. brassicae* (GLMM: *z* = 2.05, *P *= 0.040), and was marginally significant for *M. persicae* (GLMM: *z* = 1.90, *P *= 0.058). In contrast, folivory did not influence the preference of syrphid flies, and they landed as frequently on infested plants as they did on uninfested plants (Fig. 1, GLMM: *z* = 1.00, *P *= 0.322). This was indeed the case for plants infested with *B. brassicae* (GLMM: − *z* = 1.35, *P *= 0.176), and *M. persicae* (GLMM: *z* = 0.77, *P *= 0.443). However, syrphid flies landed more frequently on plants infested with *L. erysimi* compared with uninfested plants (GLMM: *z* = 2.39, *P *= 0.017). For most treatments, herbivore infestation and feeding site had the same effect on the duration of visitation and the number of flowers visited as for the landing preference for syrphid flies (Fig. 1). Syrphid flies spent more time per flower on flower-infested plants when compared with uninfested plants (Fig. 1, GLMM:* df* = 1, *χ*^2^ = 4.72, *P *= 0.030). This was recorded for plants infested with *L. erysimi* (GLMM:* df* = 1, *χ*^2^ = 10.77, *P *= 0.001), marginally significant for *M. persicae* (GLMM:* df* = 1, *χ*^2^ = 3.57, *P *= 0.059), but not for plants infested with *B. brassicae* (GLMM:* df* = 1, *χ*^2^ = 2.49, *P *= 0.115). Syrphid flies spent similar amounts of time per flower when offered a choice between flowers from leaf-infested and uninfested plants (Fig. 1, GLMM:* df* = 1, *χ*^2^ = 2.27, *P *= 0.132). However, infestation with *L. erysimi* influenced their choice. Syrphid flies spent more time per flower of plants infested with *L. erysimi* on leaves than on uninfested plants (GLMM:* df* = 1, *χ*^2^ = 4.61, *P *= 0.032). In general, visitation of syrphid flies on infested plants was not affected by aphid abundance (Table S1), except for visitation duration on flower-infested plants with *L. erysimi* (*τ* = − 0.27, *z* = − 2.43, *P* = 0.015).

**Fig. 2:**
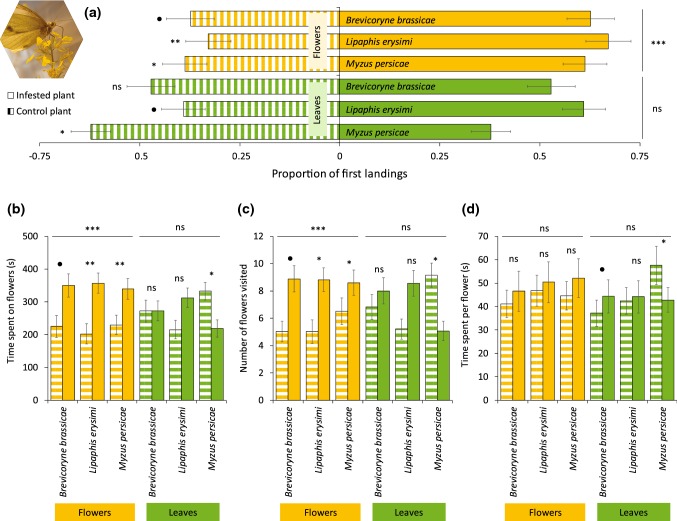
Preference of the butterfly *Pieris brassicae* for uninfested *Brassica nigra* plants or plants infested with herbivores on leaves or flowers. **a** Proportion of butterflies (Mean ± SE) that first landed on flowers or leaves of *B. nigra* plants infested with herbivores on leaves or flowers, or uninfested plants. **b** Visitation duration (Mean ± SE), **c** number of flowers visited (Mean ± SE), (**d**) and time spent per flower (Mean ± SE) by individual pollinators on infested or uninfested *B. nigra* plants. Number of replicates per herbivore treatment varied between 67 and 98 butterflies, and 7 and 10 plant pairs. Asterisks above bars indicate significant differences with *** = *P* < 0.001, ** = 0.001 ≥ *P* < 0.01, * = 0.01 ≥ *P *≤ 0.05, and ● = 0.05 > *P* < 0.1, based on Tukey’s post hoc tests. Picture shows a *P. brassicae* butterfly visiting flowers of *B. nigra*. Photograph credits: Quint Rusman

Overall, butterflies landed more frequently on flower-infested plants when compared with uninfested plants (Fig. 2, GLMM: *z* = 3.95, *P *< 0.001). This was true for plants infested with *L. erysimi* (GLMM: *z* = 2.81, *P *= 0.005) and *M. persicae* (GLMM: *z* = 2.00, *P *= 0.046), and only marginally significant when regarding *B. brassicae* (GLMM: *z* = 1.76, *P *= 0.079). Overall, folivory did not influence butterfly choice, and butterflies landed as frequently on leaf-infested plants as they did on uninfested plants (Fig. 2, GLMM: *z* = − 0.02, *P *= 0.981). This was indeed recorded when considering only plants infested with *B. brassicae* (GLMM: *z* = 0.367, *P *= 0.714), or *L. erysimi* (GLMM: *z* = 1.66, *P *= 0.096). However, butterflies landed less frequently on plants infested with *M. persicae* compared with uninfested plants (GLMM: *z* = − 2.40, *P *= 0.016). For most treatments, herbivore infestation and feeding site had the same effect on the duration of visitation and the number of flowers visited as for the landing preference for butterflies (see Fig. 2). Butterflies spent similar amounts of time per flower irrespective of the herbivore species or feeding site in plant treatments, except for plants infested with *M. persicae* on the leaves. In the latter case, butterflies spent more time per flower on uninfested plants when compared with infested plants (GLMM:* df* = 1, *χ*^2^ = 6.14, *P *= 0.013). In general, visitation of butterflies on infested plants was not affected by aphid abundance (Table S1), except for time spent per flower on leaf-infested plants with *L. erysimi* (cor = − 0.29, *t* = − 2.22, *P* = 0.031).

### Herbivore preference for leaves or flowers

**Fig. 3:**
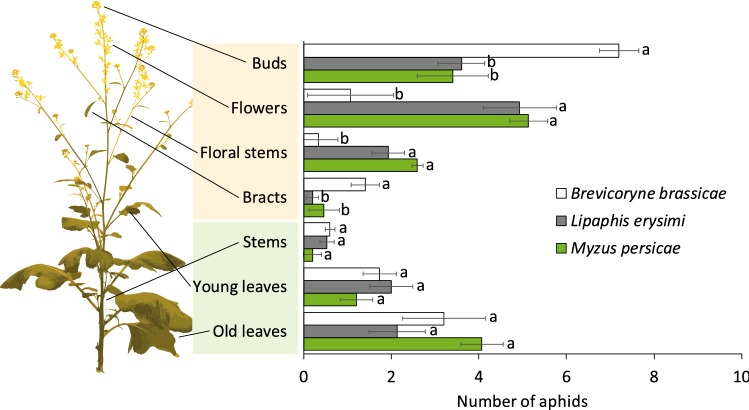
Number of winged aphids of three different species (Mean ± SE) on various plant organs of *Brassica nigra* plants. The position of 20 winged aphids was determined 24 h after release. Number of plant replicates was 15 for each aphid species. Letters indicate significant differences at *α* = 0.05 based on Tukey’s post hoc tests when comparing differences between species within a plant organ. Photograph credits: Dani Lucas-Barbosa

All three aphid species were recorded more frequently on inflorescences than on vegetative tissues (Fig. 3, Tukey’s post hoc tests; *B. brassicae*: *P *< 0.001, *L. erysimi*: *P *< 0.001, *M. persicae*: *P *< 0.001). Behavioral choices, for various plant organs within either inflorescence—buds, flowers, floral stems, bracts—or vegetative parts—stems, young leaves, old leaves—differed depending on the aphid species (Fig. 3, GLM: *χ*^2^ = 83.42,* df* = 6, *P *< 0.001). Overall, most winged aphids were recorded among buds (Fig. 3, GLM: *χ*^2^ = 141.77,* df* = 12, *P *< 0.001). When comparing the different species, relatively more winged *B. brassicae* aphids were recorded on buds and bracts compared with numbers of *L. erysimi* (Tukey’s post hoc tests, buds: *P *= 0.012; bracts: *P *= 0.007) and *M. persicae* (Tukey’s post hoc tests, buds: *P *= 0.001; bracts: *P *= 0.029). Fewer winged aphids of *B. brassicae* were found on flowers and floral stems compared with numbers of *L. erysimi* (Tukey’s post hoc tests, flowers: *P *< 0.001; floral stems: *P *= 0.001) and *M. persicae* (Tukey’s post hoc tests, flowers: *P *< 0.001; floral stems: *P *< 0.001).

### Herbivore performance on leaves or flowers

**Fig. 4:**
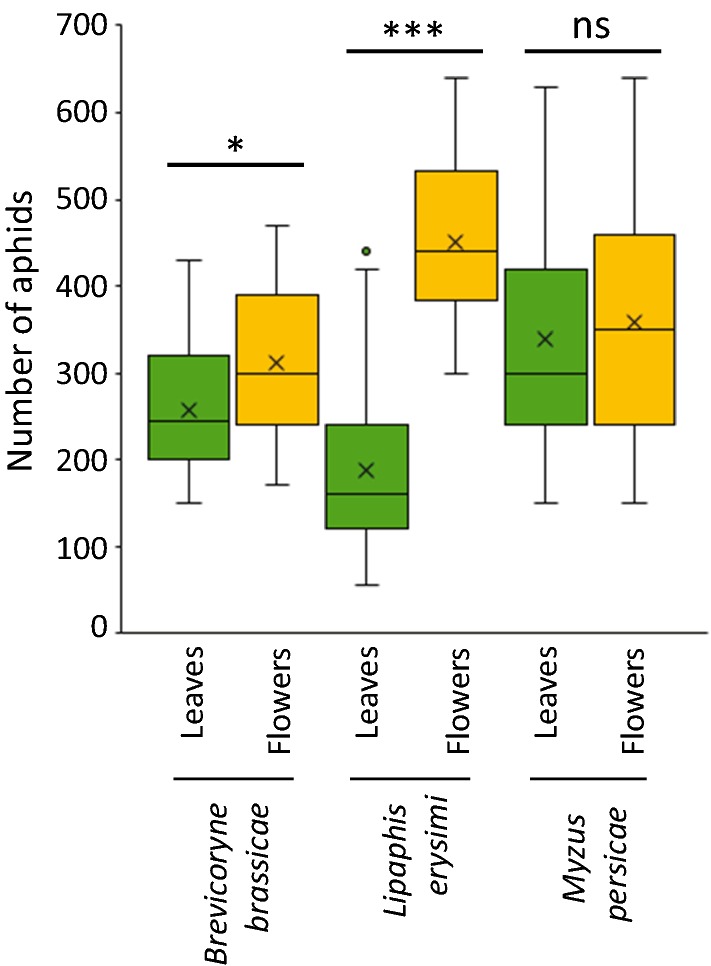
Numbers of aphids of three different species on leaves and flowers of *Brassica nigra* plants. Adult aphids and nymphs were counted 7 days after infestation. Boxplots show median (line), mean (x), first and third quartiles, minimum and maximum. Outliers are represented by circles (1.5 times the interquartile range below the first or above the third quartile). Asterisks above lines indicate significant differences between feeding sites for each aphid species based on Tukey’s post hoc tests, where * = 0.01 ≤ *P* ≤ 0.05 and *** = *P* ≤ 0.001. Number of plant replicates varied between 23 and 25

Aphid performance was affected by feeding site (GLM: *χ*^2^ = 33.89,* df* = 1, *P *< 0.001) and aphid species, resulting in a significant interaction between these two factors (GLM: *χ*^2^ = 39.83,* df* = 2, *P *< 0.001). Overall, aphids performed better on flowers than on leaves (Fig. 4, Tukey’s post hoc test, *P *= 0.041). This was the case for *B. brassicae* and *L. erysimi* (Tukey’s post hoc tests, *P *= 0.041 and *P *< 0.001, respectively), whereas *M. persicae* performed equally well on both plant tissues (Tukey’s post hoc test, *P *= 0.543). The magnitude of effect of feeding site was strongest for *L. erysimi*, for which we found 2.4 times more individuals on flowers than on leaves.

## Discussion

Our data show that indirect plant-mediated interactions between herbivores and pollinators are dependent on the feeding site chosen by herbivores. Florivory positively affected pollinator visitation, independent of the pollinator or aphid species tested. In contrast, folivory had limited effects on pollinator visitation. Flowering plants might experience florivory more often than folivory because all three herbivore species preferred to settle on flowers over leaves. The choice of feeding site matched the performance for most herbivores; the two specialist aphids performed better on flowers than on leaves, whereas the generalist aphid performed equally well on both plant parts. Taken together, the choice of the feeding site by the adult herbivores maximizes its performance and had profound impact on the outcome of plant-mediated interactions between herbivores and pollinators. Hence, the evolution of antagonist feeding behavior might affect mutualistic network assembly via trait-mediated interactions.

The importance of herbivore feeding site as determinant of plant-mediated interactions can be explained by differences in plant responses to herbivores that chose different feeding sites. Indeed, plants respond differently to the same herbivore species when feeding on distinct plant tissues, such as leaves and flowers (Farré-Armengol et al. 2015), leaves and roots (Barber et al. 2011; Hladun and Adler 2009), or leaves of different ages (Bingham and Agrawal 2010; Quintero and Bowers 2011). This is most likely caused by tissue-specific plant responses (Chrétien et al. 2018; Rusman et al. 2019a). Alternatively, plants may respond differently to different densities of aphids, which can subsequently affect plant-mediated interactions with other organisms (Kroes et al. 2017; Pineda et al. 2017). Our results suggest tissue-specific rather than density-dependent plant responses to herbivory: direct correlations between aphid numbers and pollinator visitation parameters were absent in most cases, and *M. persicae* performed equally well on flowers and leaves while having different effects on pollinator visitation. Hence, the importance of herbivore feeding site as determinant for trait-mediated interactions is likely mediated by plant responses that are specific when it comes to the tissue that is attacked (Rusman et al. 2019a; Utsumi and Shefferson 2015).

Indirect trait-mediated mechanisms suggest that herbivore-induced changes in flower traits differ when the plant is attacked by florivores or folivores, and that such changes are exploited by pollinators during foraging. Herbivore attack can induce changes in multiple flower traits at the same time, including flower size, morphology, color, volatiles, and rewards (Rusman et al. 2019b). Pollinators exploit multiple of these traits when foraging for nectar and pollen (Barragán-Fonseca et al. 2019; Junker and Parachnowitsch 2015), and hence herbivore-induced changes in flower traits have contrasting effects on pollinator visitation (Hoffmeister et al. 2016; Moreira et al. 2019; Rusman et al. 2019b). Inducible plant resistance mechanisms against florivores and folivores can influence the outcome of herbivore–pollinator interactions and differ in sign and strength depending on the feeding site chosen by the herbivore. In turn, these plant-mediated interactions between herbivores and pollinators affect plant seed production. Under herbivore attack, plants may safeguard reproduction by enhancing pollinator attraction and subsequently accelerate seed production (Lucas-Barbosa et al. 2013; Rusman et al. 2018). The increased attraction of pollinators may also be a consequence of linkage between traits involved in defense and reproduction (Jacobsen and Raguso 2018; Lucas-Barbosa 2016; Rusman et al. 2019a). Flower volatiles and pigments are involved in defense as well as reproduction and mediate interactions with mutualists and antagonists (Johnson et al. 2015; Lucas-Barbosa et al. 2011). Herbivore-induced changes in the volatile profile of flowering plants include enhanced emission of compounds that are attractive to natural enemies of herbivores (Schiestl et al. 2014), and these compounds can also be attractive for pollinators (Knauer et al. 2018). Pigments that color the flower such as flavonoids also serve as repellent/toxic compounds against herbivores (Gronquist et al. 2001). Hence, herbivore-induced changes in these compounds, to for instance, increase plant resistance to attack (Boyer et al. 2016), could also enhance the visual signal of flowers for pollinators. Indeed, folivory and florivory have different effects on flower volatiles (Farré-Armengol et al. 2015), and we expect similar differences for effects on flower color. Florivory rather than folivory may thereby induce changes in flower traits that increase the apparency of infested plants to foraging pollinators. Flower volatiles and flower color can also indicate flower rewards status (Gómez et al. 2008; Haverkamp et al. 2016; Raine and Chittka 2007). Florivore-induced changes in flower traits may promise naïve pollinators more or higher quality food as compared to folivore-induced changes. In addition, flower feeding by aphids might actually increase nectar quality. Aphids are known to change sink-source dynamics in the plant (Jakobs et al. 2019; Züst and Agrawal 2016). Aphid feeding could increase the sink strength of infested flower heads for nutrients including nitrogen, and thereby increase nectar quality. Taken together, changes in traits as part of the plants defensive response that is fine-tuned to deal with herbivores feeding on flowers may yield plants more attractive for pollinators.

Although herbivore-induced changes in flower traits are explaining the outcome of herbivore–pollinator interactions, we cannot fully exclude other indirect or direct mechanisms. Florivore-induced changes in pollinator attraction might be mediated indirectly by interaction modification (Wootton 1994): florivore presence can change how flower traits are perceived by flower visitors without affecting the trait itself. In our study, the presence of the aphids could have enhanced the visual appearance of the flowers, by enhancing contrast with the background, leading to an increase in pollinator attraction. Alternatively, the aphids themselves could have attracted the pollinators. Aphids can attract pollinators with their honeydew production, or syrphid flies specifically because their larvae feed on aphids (Almohamad et al. 2009; Meiners et al. 2017). However, most inflorescences of herbivore-infested plants remained free of herbivores, and we never observed that pollinators were particularly attracted or repelled by herbivore-infested inflorescences or the presence of the herbivores themselves in the greenhouse or field (pers. obs. Quint Rusman and Peter Karssemeijer). Since effects of florivory had positive effects on pollinators, we think that avoidance mechanisms do not play a role here (Lohman et al. 1996; Moreira et al. 2019). Thus, the herbivore–pollinator interactions observed in this study were most likely mediated by changes in flower traits.

Our study shows that the outcome of herbivore–pollinator interactions depends on the feeding site chosen by the herbivore. Such plant-mediated herbivore–pollinator interactions may cascade to affect pollinator community assembly and plant reproduction (Chautá et al. 2017; Hoffmeister et al. 2016; Rusman et al. 2018). Herbivore-induced changes in pollinator community composition and plant reproduction likely impose selection on flower traits and plant defense (Johnson et al. 2015; Lucas-Barbosa 2016; Poelman and Kessler 2016), which could lead to the rapid evolution of plant traits (Gervasi and Schiestl 2017; Schiestl and Johnson 2013). Changes in plant traits may in turn affect the feeding preference of herbivores (McCall and Irwin 2006; McCall et al. 2013; Strauss and Whittall 2006). Specificity of plant responses to the feeding site chosen by herbivores can thereby not only influence the evolution of herbivore feeding behavior, but also of plant defense and reproduction (Ohgushi 2016; Poelman and Kessler 2016), and determine the linkage between antagonistic and mutualistic plant-associated communities (Rusman et al. 2019a). Understanding the dynamic interplay between ecology and evolution is critical for our understanding of complex species interactions in plant-associated communities.

## Electronic supplementary material

Below is the link to the electronic supplementary material.

Supplementary material 1 (PDF 183 kb)
