# Supplementary material for: Settling on leaves or flowers: herbivore feeding site determines the outcome of indirect interactions between herbivores and pollinators
Source: Oecologia. 2019 Nov 4;191(4):887–96. doi: 10.1007/s00442-019-04539-1 (PMC6854048; doi:10.1007/s00442-019-04539-1)
Supplement: Supplementary file 1 — Supplementary material 1 (PDF 183 kb) [file 442_2019_4539_MOESM1_ESM.pdf]

Supporting Information

Settling on leaves or flowers: herbivore feeding site determines the outcome of indirect interactions between herbivores and pollinators

Quint Rusman, Peter N. Karssemeijer, Dani Lucas-Barbosa, and Erik H. Poelman

Corresponding author: quint.rusman@hotmail.com

Table S1. Effect of aphid abundance on visitation of the butterfly *Pieris brassicae* and syrphid fly *Episyrphus balteatus* on *Brassica nigra* plants infested with aphids on leaves or flowers. Correlation coefficient (cor) was computed using the *Pearson* or *Kendall* method, depending on the distribution of the data. For aphids on leaves + flowers we combined the data for aphids on either leaves or flowers. Bold values indicate results where $P \leq 0.05$. Italic values indicate results where $P \leq 0.1$.

	Brevicoryne brassicae Leaves + flowers			*B. brassicae* Leaves			*B. brassicae* Flowers			*Lipaphis erysimi* Leaves + flowers			*L. erysimi* Leaves			*L. erysimi* Flowers			*Myzus persicae* Leaves + flowers			*M. persicae* Leaves			*M. persicae* Flowers		
	cor	z / t	*P*	cor	z / t	*P*	cor	z / t	*P*	cor	z / t	*P*	cor	z / t	*P*	cor	z / t	*P*	cor	z / t	*P*	cor	z / t	*P*	cor	z / t	*P*
Butterfly visitation																											
Visitation time	0.09	1.20	0.230	0.07	0.46	0.646	-0.02	-0.19	0.850	0.02	0.19	0.846	-0.06	-0.43	0.666	-0.03	-0.21	0.834	0.08	1.07	0.283	-0.11	-0.72	0.475	0.24	1.76	*0.085*
Number of flowers visited	-0.06	-0.57	0.569	-0.10	-0.62	0.536	-0.04	-0.24	0.813	-0.03	-0.34	0.733	0.04	0.27	0.786	-0.12	-0.88	0.385	-0.09	-0.89	0.374	-0.12	-0.81	0.420	-0.10	-0.72	0.476
Time spent per flower	0.09	1.23	0.220	0.16	1.03	0.311	-0.03	-0.24	0.814	-0.04	-0.61	0.545	-0.29	-2.22	**0.031**	0.22	1.56	0.125	0.11	1.60	0.109	-0.13	-0.85	0.398	0.21	1.53	0.133
Syrphid fly visitation																											
Visitation time	0.02	0.24	0.814	0.04	0.29	0.774	0.06	0.55	0.579	-0.10	-1.30	0.195	0.11	0.71	0.479	-0.27	-2.43	**0.015**	0.09	1.25	0.211	0.20	1.37	0.177	0.10	0.92	0.356
Number of flowers visited	0.12	-1.08	0.282	-0.05	-0.30	0.765	-0.19	-1.22	0.228	0.08	0.71	0.480	-0.07	-0.42	0.674	0.05	0.31	0.759	0.13	1.32	0.191	0.06	0.380	0.706	0.23	1.60	0.117
Time spent per flower	0.11	1.31	0.190	-0.05	-0.26	0.800	0.04	0.25	0.807	-0.07	-0.90	0.370	0.01	0.08	0.934	-0.18	-1.18	0.247	-0.04	-0.63	0.532	0.01	0.108	0.914	-0.09	-0.59	0.558
